# Modified skills of trypsin-digested retinal vasculature mount preparation and string vessels observation

**DOI:** 10.1186/s12886-026-04951-1

**Published:** 2026-05-25

**Authors:** Mi Song, Haoyu Li, Benteng Ma, Baihua Chen

**Affiliations:** 1 Department of Ophthalmology, The Second Xiangya Hospital, Central South University, No.139 Middle Renmin Road, Changsha, Hunan 410011 China; 2 Hunan Clinical Research Center of Ophthalmic Disease, No.139 Middle Renmin Road, Changsha, Hunan 410011 China

**Keywords:** Retinal vasculature mount, Trypsin digestion, String vessel, Interpapillary bridge, Acellular capillary, Angiogenesis

## Abstract

Trypsin-digested retinal vasculature mount is a key method to study vascular retinopathy. However, its failure rate and difficulty maintaining the vascular network’s integrity limit its wide application. String vessels occur in the vasculature of different tissues in many species. However, the definition and function of string vessels are full of controversy. This study aimed to explore the skills of preparing complete and repeatable retinal vasculature mounts and further understand the string vessel through observation. We found that the integrity of the retinal vascular network was successfully protected by using toothless micro forceps to clamp the internal limiting membrane and using glass rods to lift the vascular network from the water surface. This modification will not waste the hard-won samples. We observed the different states of the string vessels and hypothesized that they might participate in angiogenesis during vascular development or under pathological conditions.

**Supplementary Information:**

The online version contains supplementary material available at 10.1186/s12886-026-04951-1.

## Introduction

Diabetic retinopathy and retinopathy of prematurity are common vascular retinopathies and are two important causes of visual impairment [1, 2]. Previous studies have established various diabetic retinopathy models [3] and OIR (oxygen-induced retinopathy) models [4, 5] to explore vascular retinopathy’s pathological mechanism and treatment. However, due to the long feeding period of the diabetic retinopathy model and the difficulty in obtaining samples, experiments with high success rates are needed to study the samples. The trypsin-digested retinal vascular mount is important for studying retinal vessels [6, 7]. However, given the technical difficulties in preparing, obtaining a high-quality retinal vascular network mount is often challenging, affecting the observation of experimental results and limiting the widespread application of this practical method [8]. Kuwabara and Cogan first proposed Trypsin-digested retinal vasculature mount preparation in 1960 [9].Subsequently, researchers modified the preparation method. Some literature does not provide detailed methodological descriptions; Laver and colleagues used purified elastase to digest the retina, but did not display the complete retinal vascular mount image [8]. Dietrich and colleagues cut the retina into quarters and cleaned it with the syringe after mounting [10]. However, it is still has challenging to thoroughly clean the nerve fiber tissue between the vascular network and the vascular easily adheres to the instruments and takes off from the slide. Chou et al. [11] have made outstanding contributions in improving mouse retinal vasculature mount preparation. However, according to their methods, obtaining complete, fully unfolded, clean, clear, and high success rate retinal vascular mounts is still tricky. Herein, we hope that through method exploration and experience summary, we can prepare a complete and beautiful retinal vascular mount with a high success rate. If the researchers master the techniques, they may study vascular retinopathy at a low cost.

String vessels are found in the blood vessels of various tissues in many species [12]. The string vessel has many other names, including acellular capillary, intercapillary bridge, and intervascular strand. In ophthalmology, acellular capillary and intercapillary bridges are usually used to describe string vessels. However, the definition of acellular capillaries varies in different research. Some researchers define it as a capillary-sized tube with no nucleus anywhere along its length [13, 14]. Some researchers excluded tubes with diameters less than 20% of the diameter of adjacent capillaries [15]. Some researchers believe capillaries without pericytes and endothelial cells are considered acellular capillaries [16, 17]. Some studies conflated the definitions of acellular capillary and intercapillary bridges. We believe that defining an acellular capillary solely according to the pipe diameter is inappropriate without a complete understanding of its function. The string vessel’s nature and function are also controversial. Ramon believed that the string vessels might be residual connective tissue fibers formed by narrowed lumens, insufficient blood flow, and vascular atrophy [18]. However, Challa discovered string vessels in normal human cerebral blood vessels, ranging from premature infants to the elderly [19]. Mendes [20] reported that intervascular bridging cells are a special pericyte subtype that shares characteristics with endothelial cells, which can help regulate vasodilation and contraction and maintain stable circulation. Our study tries to distinguish the acellular capillary and intercapillary bridge. Furthermore, we aimed to understand the string vessels further through observation.

## Methods and materials

### Trypsin-digested retinal vasculature mount preparation

#### Reagents and instruments

**Fig. 1:**
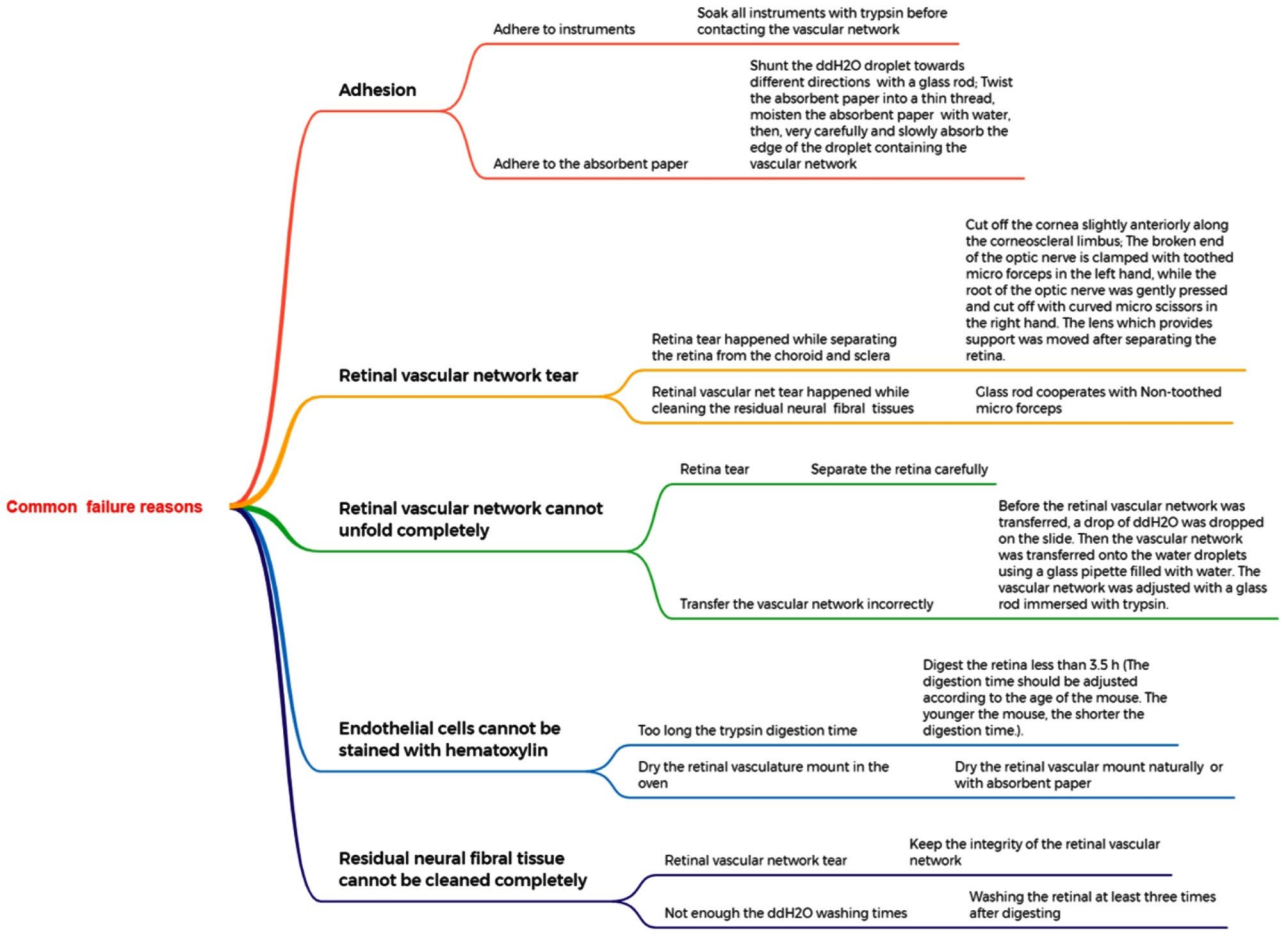
Common failure reasons analysis of trypsin-digested retinal vasculature mount preparation

Constant temperature water bath (Tester, China); Anatomical microscope (Leica, Germany); Shaker (SCILOGEX, USA); 4% paraformaldehyde solution (ServiceBio, China); PBS solution without calcium and magnesium (Solarbio, China); 3% trypsin powder (1:250) (Solarbio, China); PAS staining kit (Solarbio, China) (S Fig. 1).

### Steps

#### Eyeball enucleation and fixation

Sacrifice the mice or rats by cervical dislocation. Open the eyelid with the left hand. Place the curved forceps’ two legs above and below the eyeball and gently press down to make the eyeball protrude. Close the forceps at the root of the optic nerve and extract the eyeball vertically. Fixate eyeballs in 4% paraformaldehyde solution at 4℃ for 48 h.

#### Retinal separation and ddH_2_O rinsing and immersing

**Fig. 2:**
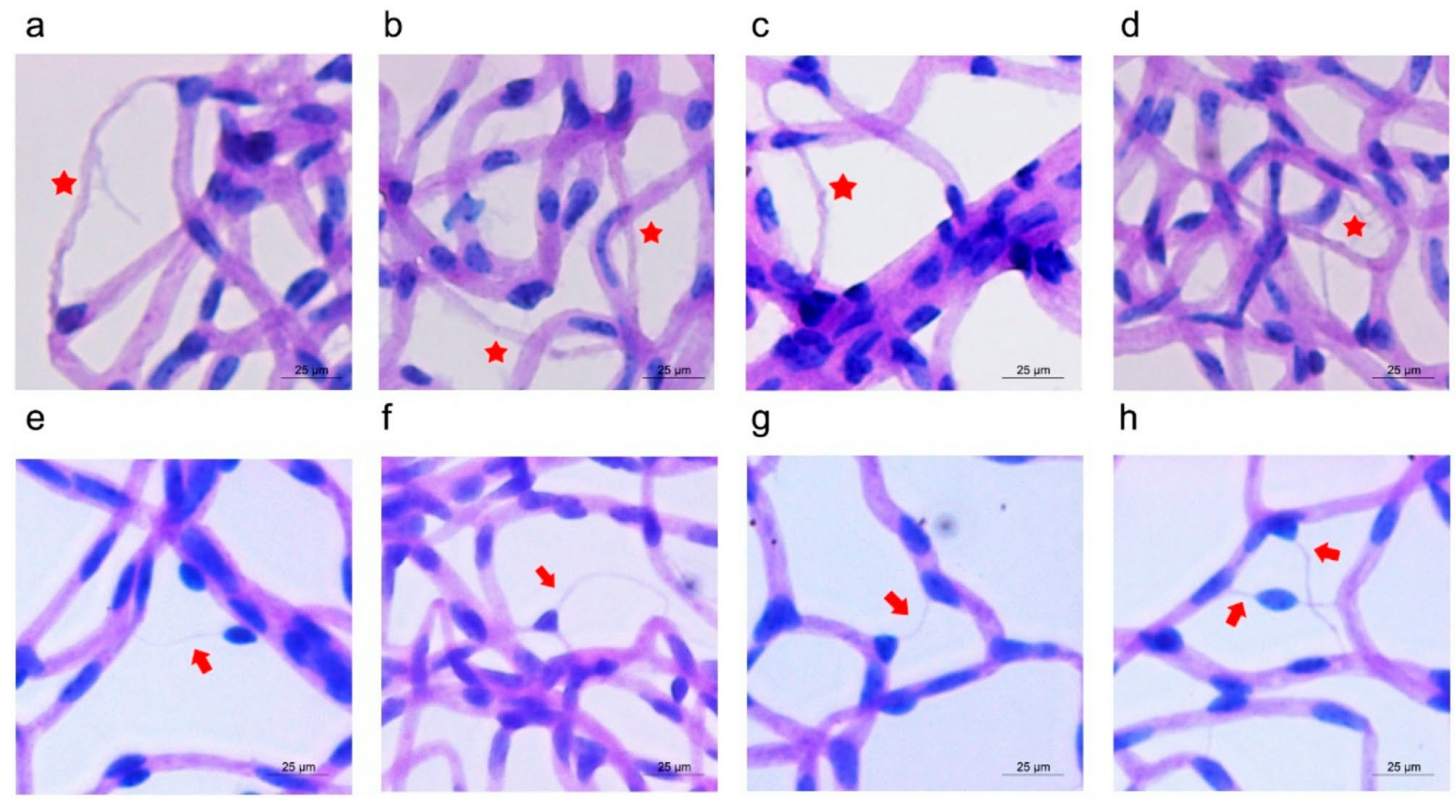
Qualitative morphological comparison of acellular capillary-like structures and intercapillary bridge-like structures. The red star indicates an acellular capillary-like structure; the red arrow indicates an intercapillary bridge-like structure. **a**–**d** Representative acellular capillary-like structures observed in PAS-stained trypsin-digested retinal vasculature mounts from STZ-induced diabetic mice (*n* = 6 mice). These structures appeared relatively wider, lacked visible nuclei along their length, and showed irregular or pleated edges. **e**–**h** Representative intercapillary bridge-like structures observed in PAS-stained trypsin-digested retinal vasculature mounts from normal mice (*n* = 6 mice). These structures appeared thinner, had smooth edges, and were often located near or between vascular-associated nuclei or adjacent capillaries. These images represent qualitative morphological observations; formal quantitative morphometric analysis was not performed

**Fig. 3:**
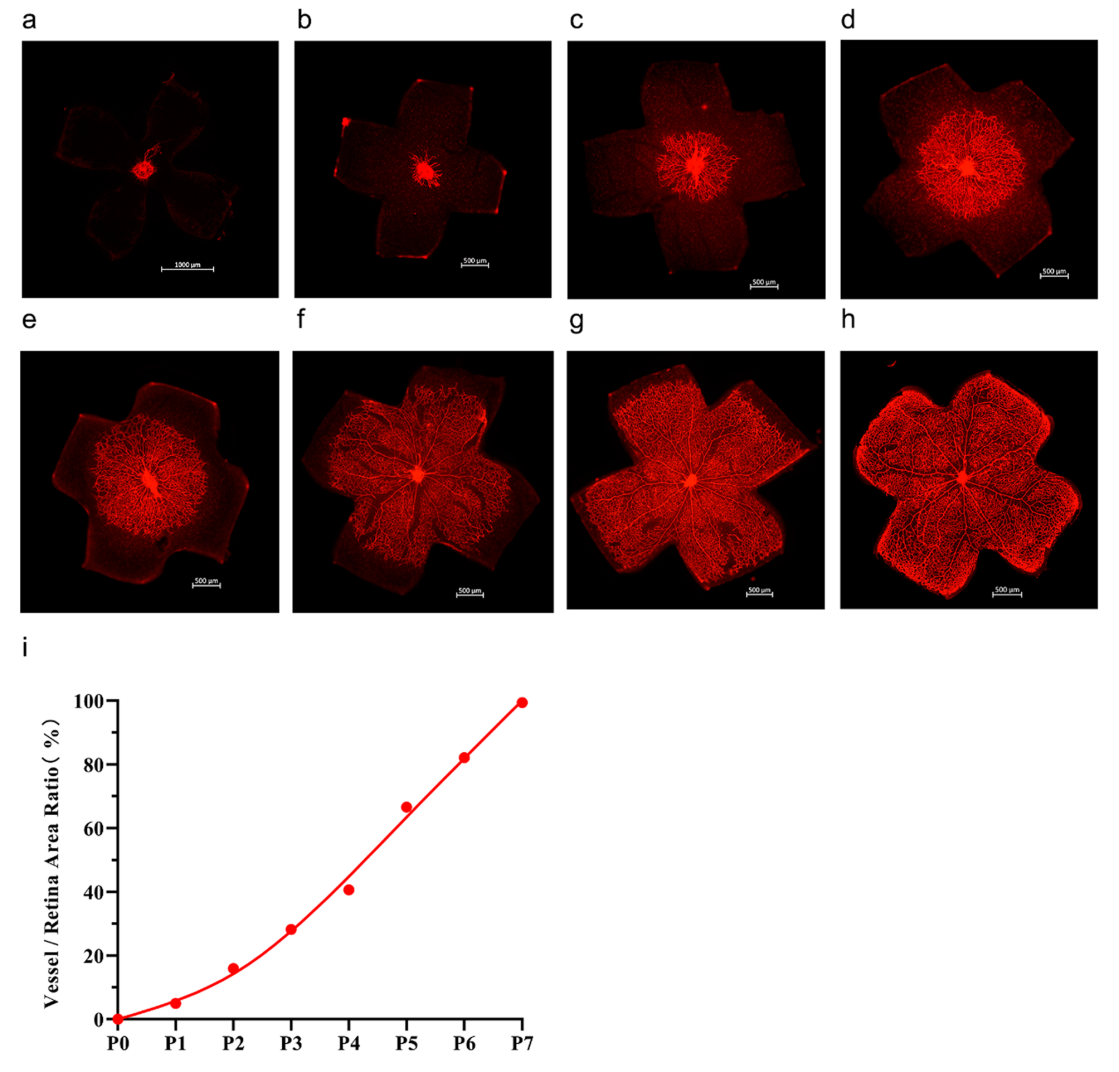
Postnatal development of the mouse retinal vasculature visualized by IB4-stained retinal whole-mounts. **a–h** Representative retinal whole-mount fluorescence images showing the progressive development of the superficial retinal vascular network from postnatal day 0 to postnatal day 7. **a** At P0, retinal vascular staining is minimal and limited to the central optic-disc region. **b–d** From P1 to P3, the vascular plexus begins to sprout radially from the optic nerve head and extends toward the mid-peripheral retina. **e–g** From P4 to P6, the vascular network expands progressively, with increasing peripheral extension, branching density, and vascular coverage. **h** At P7, the superficial vascular plexus has extended close to the retinal periphery and forms an almost complete retinal vascular network. **i** Quantitative analysis of the vascularized retinal area ratio from P0 to P7, demonstrating a gradual increase in retinal vascular coverage during postnatal development. Scale bars are shown in each panel

Cut off the muscle and adipose tissues attached to the eyeball. Make a round-like incision in the center of the cornea with a sharp surgical blade and curved micro scissors. Then, the cornea was cut along slightly anterior to the corneoscleral limbus with curved micro scissors. Remove only the iris; The lens is temporarily retained to support the retina and avoid collapse. The optic nerve is clamped with a toothed forceps in the left hand. The optic nerve is clipped with curved micro scissors in the right hand, with slightly pressing against the root of the optic nerve (to separate the retina from the choroid-sclera at the root of the optic nerve), the eyecup was moved to another petri dish filled with ddH_2_O. The choroid-sclera layer was cut into three pieces with curved micro scissors. The retina was gradually and carefully separated from the choroid-sclera layer by non-toothed micro forceps. When separate to the optic nerve root, use toothless forceps to hold the choroid-sclera layer, and push the retina away. Then, the lens is removed. The retina was transferred to a 24-well plate with ddH_2_O, using a large-diameter plastic Pasteur pipette filled with ddH_2_O. Wash the retina with ddH_2_O on a 90PRM shaker five times, 1 h each time. The ddH_2_O was sucked out with a plastic Pasteur tube, and fresh ddH_2_O was added. The retinas were immersed at room temperature for 36 h (S Figs. 2 and 3).

#### Trypsin digestion and ddH_2_O washing

Add 3% trypsin solution to each well of the 24-well plate and place in a constant temperature water bath at 37℃ for digestion for 3 h. After digesting, the retina was washed with the ddH_2_O twice on a 140 PRM shaker, 5 min each time, and 15 min for the last time. The Pasteur pipette was used to suck the ddH_2_O, and the fresh ddH_2_O was added. (This step is also crucial because it can remove the outer layer of the retina, making the retina transparent.) The digestion time should be adjusted according to the age of the mouse.

#### Retinal vascular network separation

Soak all instruments that will contact the vascular network with trypsin (This operation can effectively prevent adhesion). The retinal vascular network was separated under the microscope with a black background. Use a glass pipette filled with water to suck and blow near the retina to observe the morphology of the retina and find the edge of the internal limiting membrane. The key modified skill: a non-tooth forceps in the right hand was used to gently clamp the central part of the internal limiting membrane to control the position of the retina, and a glass rod in the left hand under the retina was used to lift the retina to leave the water, and then quickly return to the water. At this moment, most of the residual nerve fiber tissue was removed, and the integrity of the vascular network will no longer be damaged. Then, move the vascular network up and down the water’s surface with non-toothed micro forceps. If it is found that the vascular network cannot be unfolded in water, the glass rod is used to unfold the vascular network. If the water becomes cloudy while cleaning the vascular network, the glass pipette filled with water transfers the vascular network to another hole for further cleaning until the remaining nerve fiber tissue cannot be seen under the microscope. (The key operation skills are shown in S Fig. 3)

#### Retinal vascular network mounting and PAS staining

A drop of clean water was placed on the slide, and the vascular network was transferred to the water drop using a glass pipette filled with water. Adjust the blood vessel network with a glass rod to unfold fully on the surface of the water droplet. The water droplet was shunted in different directions with a glass rod. The water, far away from the vascular network, was slowly absorbed by the paper, which had absorbed water and been twisted into a thin thread.

PAS staining process: When the vascular network is completely dry and firmly adhered to the glass slide, immerse the glass slide once and place it obliquely to allow it to dry quickly and naturally. Periodate oxidants for 4 min, wash them twice with tap water for 1 min each time, wash them with ddH_2_O for 1 min, dry them, then, Schiff reagent for 10 min, soak them with tap water for 10 min, and hematoxylin for 4 min, wash them with tap water for 10 min.

After dewatering and sealing with glycerin gelatin, the morphology of retinal blood vessels can be observed under a microscope. If intended to observe the morphology of retinal blood vessels before sealing, a drop of ddH_2_O can also be dropped on the vascular network, making the specimen more beautiful. After photographing and recording, the number of acellular capillaries and intercapillary bridges was counted. Do not use the oven to dry. Otherwise, it may cause the rupture of the nuclear membrane of retinal vascular endothelial cells, and endothelial cells cannot be stained with hematoxylin.

### Experimental animals

Different weeks of C57 / BL6 mouse, eight weeks of SD rats and the OIR mouse model. Experimental animals are bought from the Hunan SJA Laboratory Anima Co., Ltd, and raised in the SPF animal laboratory of the Second Xiangya Hospital. All animal procedures are approved by the Second Xiangya Hospital of Central South University’s animal ethics committee and strictly abide by the ARVO guidelines for animal experiments and research.

Diabetes model: 6 male C57BL/6j mice (8 weeks) were fasted for 24 h and injected intraperitoneally with streptozotocin (150 mg/kg, STZ dissolved in citric acid buffer, PH 4.5). The age-matched non-diabetic control group received the same amount of citric acid buffer intraperitoneal injection. After 72 h and 1 week, the rats with blood glucose > 16.7 mmol/l were defined as diabetic. One year after successful modeling, mice were sacrificed by cervical dislocation to obtain eyeball tissue.

OIR model: C57BL/6j (P7-P12) was exposed to 75% O2 for five days and then returned to 21%O_2_ for another five days. The mice were sacrificed by cervical dislocation, and the eyeball tissue was obtained.

### Retinal fluorescent whole-mount

The eyeball was enucleated, the cornea and lens were removed, the retina was separated, and the vitreous was removed. The retinal incubated with IB4 conjugated with Alexa Fluor 594 overnight, and the retina was cut quarterly, then was mounted. The whole retinal mount was sealed with anti-fluorescence quenching tablets. Zeiss microscope was used for observation and photography [21].

### String vessel quantification

PAS-stained trypsin-digested retinal vasculature mounts were imaged at 200× magnification. For each mouse, 8 non-overlapping microscopic fields were selected from comparable mid-retinal vascular regions. Each analyzed field corresponded to one full 200× microscopic image frame acquired under identical microscope and camera settings and measured approximately 0.90 mm × 0.60 mm, corresponding to 0.54 mm². Fields with tissue folding, tearing, incomplete unfolding, poor staining, or obvious preparation artifacts were excluded. String vessels were manually counted according to the morphological criteria described above. The mean value from the 8 analyzed fields of each mouse was used as one biological replicate for statistical analysis. A total of 6 mice per group/time point were included. Data are presented as mean + SD. Differences among groups were analyzed by one-way ANOVA followed by Tukey’s multiple-comparison test. Statistical significance was set at *p* < 0.05.

### Statistical analysis

SPSS, Ver.23.0 was used for statistical analysis, and GraphPad Prism, Ver.8.0 was used for plotting. The data were demonstrated as mean ± sd. The differences between groups were tested by ANOVA. *P* < 0.05 means a significant difference.

## Results

### Shortcomings of instruments used in previous

The following experiences are summarized through many attempts: a.Glass pipette. It has a limited ability to clear residual neural fibrous tissues. b. Plastic Pasteur pipette. The vascular network easily adheres to the limbus of the plastic pipette orifice. c. 200 µl tip. It has difficulty controlling the blowing and sucking force and is susceptible to damaging the integrity of the vascular network. d. Non-toothed micro forceps. It can not counter the gravity of the retina with many residual neural fibrous tissues, and cause vessels to be incomplete when used alone. e. Glass rod. It is too smooth to hold the retina when used alone. f. The modified method. The glass rod cooperates with non-toothed micro forceps, which are very suitable for separating and cleaning the retinal vascular network and have a high success rate. (Table 1)

**Table 1:** Disadvantages of instruments used to separate and clean the retinal vascular network in previous literature

Instruments	Limitations
Glass pipette	Limited to clear residual neural fibrous tissues
Plastic Pasteur pipette	Easy to adhere the vascular network to the limbus of the pipe orifice of the pipette.
200 µl tip	Difficult to control the blowing and sucking force, and easy to damage the integrity of the vascular network
Non-toothed micro forceps	Cannot counter the gravity of the retina with many residual neural fibrous tissues and cause vessels to disconnect when used alone
Glass rod	Too smooth to hold the retina when used alone
Glass rod cooperates with non-toothed micro forceps	None. Very suitable for separating and cleaning the retinal vascular network; High success rate

### Analysis of common technical problems during trypsin-digested retinal vascular network mount preparation

Representative technical problems encountered during unmodified or suboptimal preparation, together with corresponding preventive measures, are summarized as follows: (a) Adhesion. To prevent the vascular network from adhering to the operating instruments, all instruments are soaked with trypsin before contacting the vascular network; In order to prevent the vascular network from adhering to the paper: First, shunt the ddH_2_O droplet in different directions with a glass rod, and then twist the wet paper into a thin thread, and finally very carefully and slowly absorb the edges of the droplet far away from the vascular network. (b) Retinal vascular network tear. In order to prevent tearing when the retina is separated from the choroid-sclera layer, first, the cornea was cut slightly forward along the corneoscleral limbus, then the broken end of the optic nerve was clamped with toothed micro-forceps in the left hand, and the scissors in the right hand gently press the optic nerve root and cut it. The lens that supports the retina is removed after retinal separation; the cooperation of the glass rod and toothless micro forceps can prevent the retinal vascular network from tearing while removing residual nerve fiber tissue. (c) The retinal vascular network cannot be fully expanded. Since the broken retinal vascular network is not conducive to unfolding the vascular network, the integrity of the vascular network should be ensured during the whole preparation process. The correct vascular network transfer method is needed: before the retinal vascular network is transferred, drop a ddH_2_O droplet on the slide. The vascular network was then transferred onto the water droplet using a glass pipette filled with water. Adjust the vascular network with a glass rod immersed in trypsin to help it fully unfold. (d) Endothelial cell nuclei cannot be stained with hematoxylin. Too long trypsin digestion will destroy the vascular structure, so the digestion time of the retina is less than 3.5 h (the digestion time is adjusted according to the age of the mouse; The younger the mouse, the shorter the digestion time). Dry retinal vascular mounts should be avoided in an oven, which will damage the nuclear membrane. (e) The residual nerve fiber tissue cannot be wholly cleaned. The tear of the retinal vascular network is not conducive to removing the remnant. So, the integrity of the retinal vascular network must be maintained throughout the process. Complete removal of residual tissue requires the aid of water surface tension; therefore, the vascular network should be gently lifted above and returned to the water surface several times. S Fig. 7 shows representative examples of possible technical problems, including residual neural fibrous tissue/internal limiting membrane remnants, poor nuclear visualization after hematoxylin staining, and incomplete unfolding of the vascular network. These examples are intended to illustrate preventable technical issues rather than typical or unavoidable outcomes of the preparation (S Fig. 7 and Fig. 1).

### Representative images of success retinal vasculature mounts stained with PAS

**Fig. 4:**
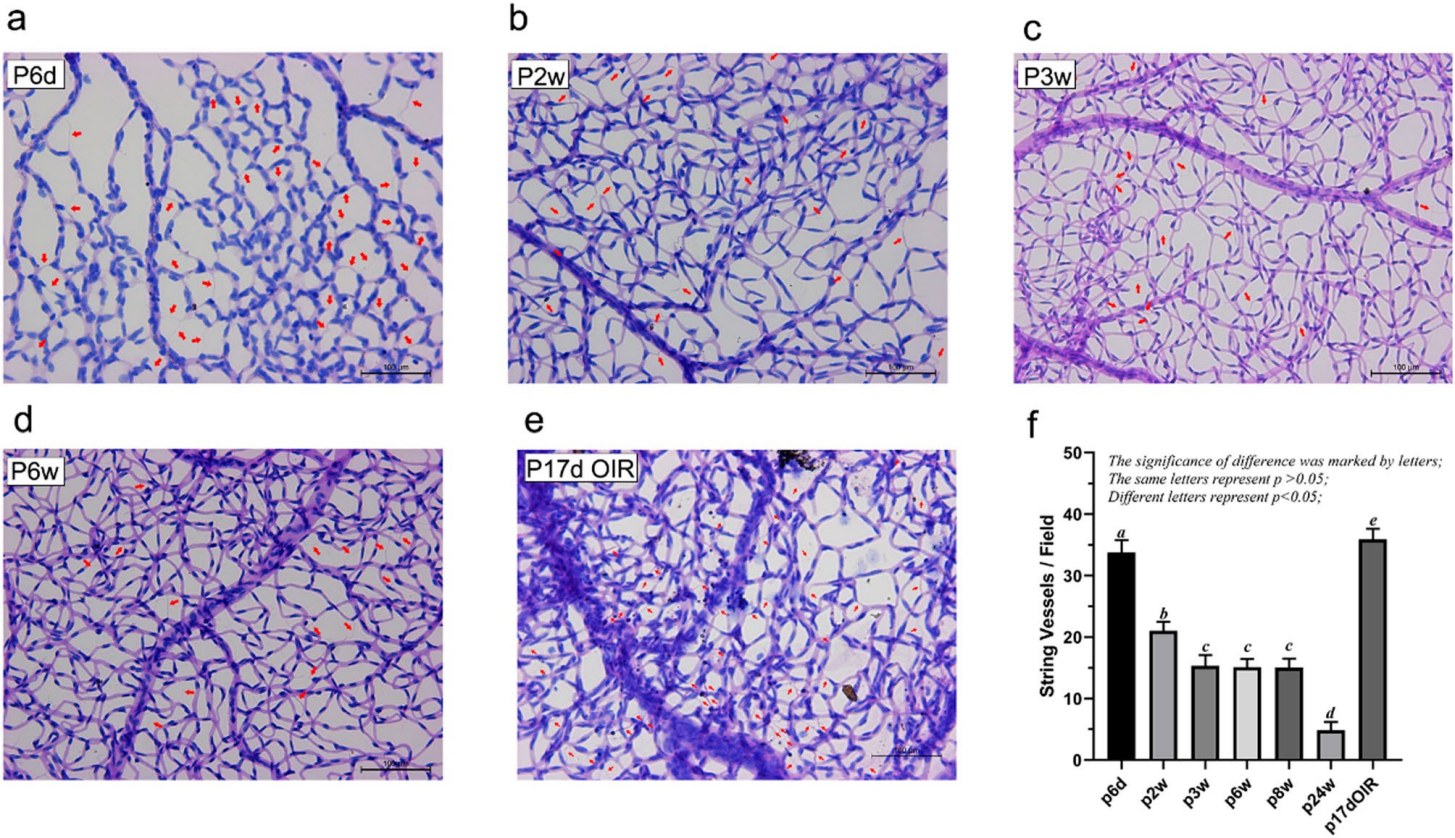
Number of string vessels at different postnatal ages and in the OIR model. Red arrows indicate representative string vessels (*n* = 6/group). **a** P6d normal C57BL/6 mouse retina. **b** P2w normal C57BL/6 mouse retina. **c** P3w normal C57BL/6 mouse retina. **d** P6w normal C57BL/6 mouse retina. **e** P17d oxygen-induced retinopathy mouse retina. **f** Quantitative comparison of string vessels per field in P6d, P2w, P3w, P6w, P8w, P24w, and P17d OIR mice. Images were captured at 200× magnification. For each mouse, 8 non-overlapping fields from comparable mid-retinal vascular regions were analyzed. Each field measured approximately 0.90 mm × 0.60 mm, corresponding to 0.54 mm². A total of 5 mice per group/time point were included. The mean value from the 8 fields of each mouse was used as one biological replicate for statistical analysis. Bars represent mean + SD; upper error bars are shown. Different letters indicate significant differences between groups, *p* < 0.05; the same letters indicate no significant difference, *p* > 0.05. Statistical analysis was performed using one-way ANOVA followed by Tukey’s multiple-comparison test

**Fig. 5:**
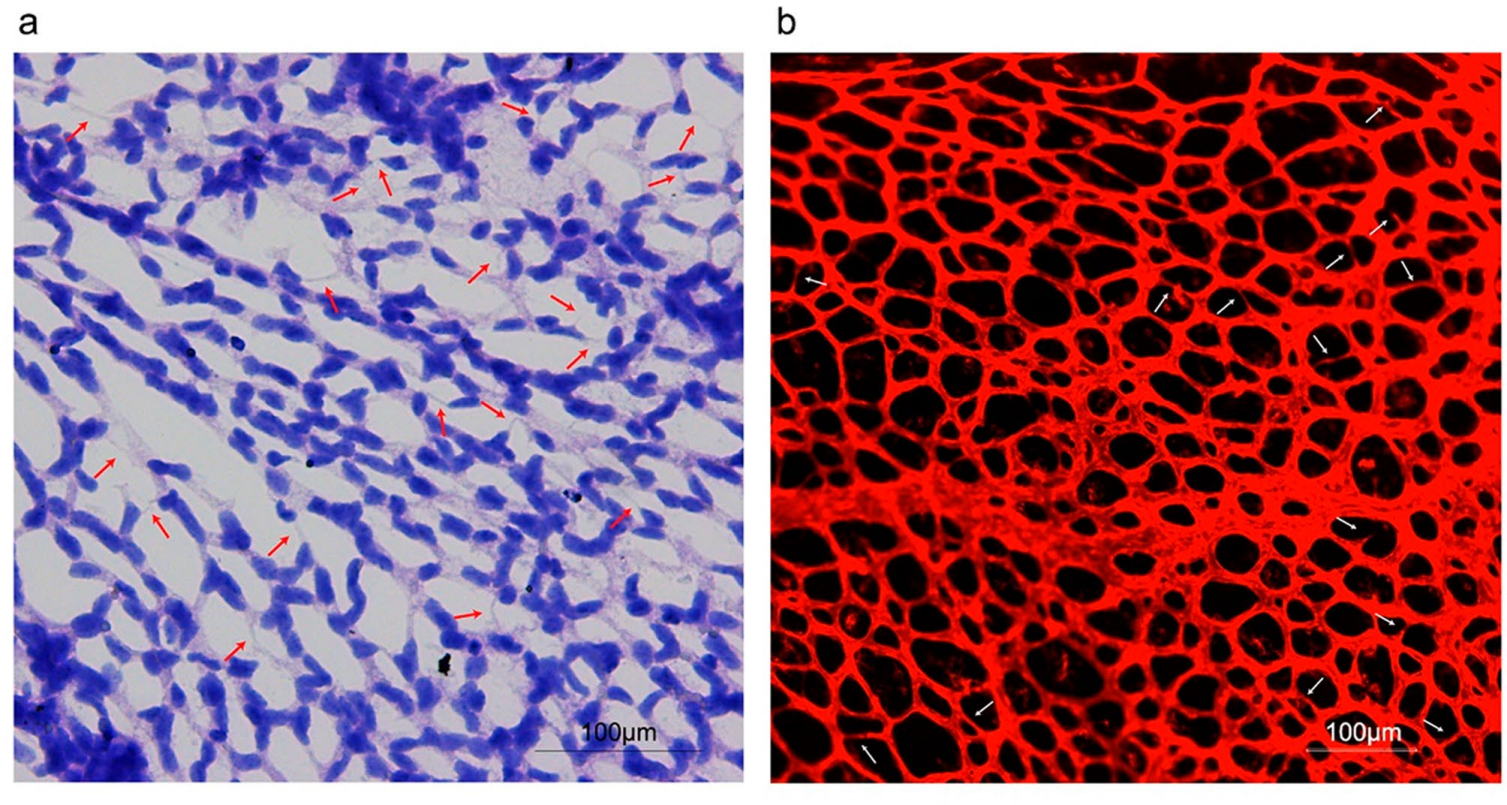
Morphology of intercapillary bridge-like structures in P6d C57BL/6 mouse retina. Representative images from P6d C57BL/6 mice (*n* = 6 mice) **a** PAS-stained trypsin-digested retinal vasculature mount showing string-vessel/intercapillary bridge-like structures and associated nuclear morphology. **b** IB4-stained retinal whole-mount showing the overall retinal vascular network. IB4 staining visualizes endothelial-associated vascular structures but may not detect endothelium-free acellular capillaries or basement-membrane remnants. Therefore, this figure illustrates the technical difference between the two preparations rather than a direct comparison of their sensitivity for detecting all string-vessel-like structures

**Fig. 6:**
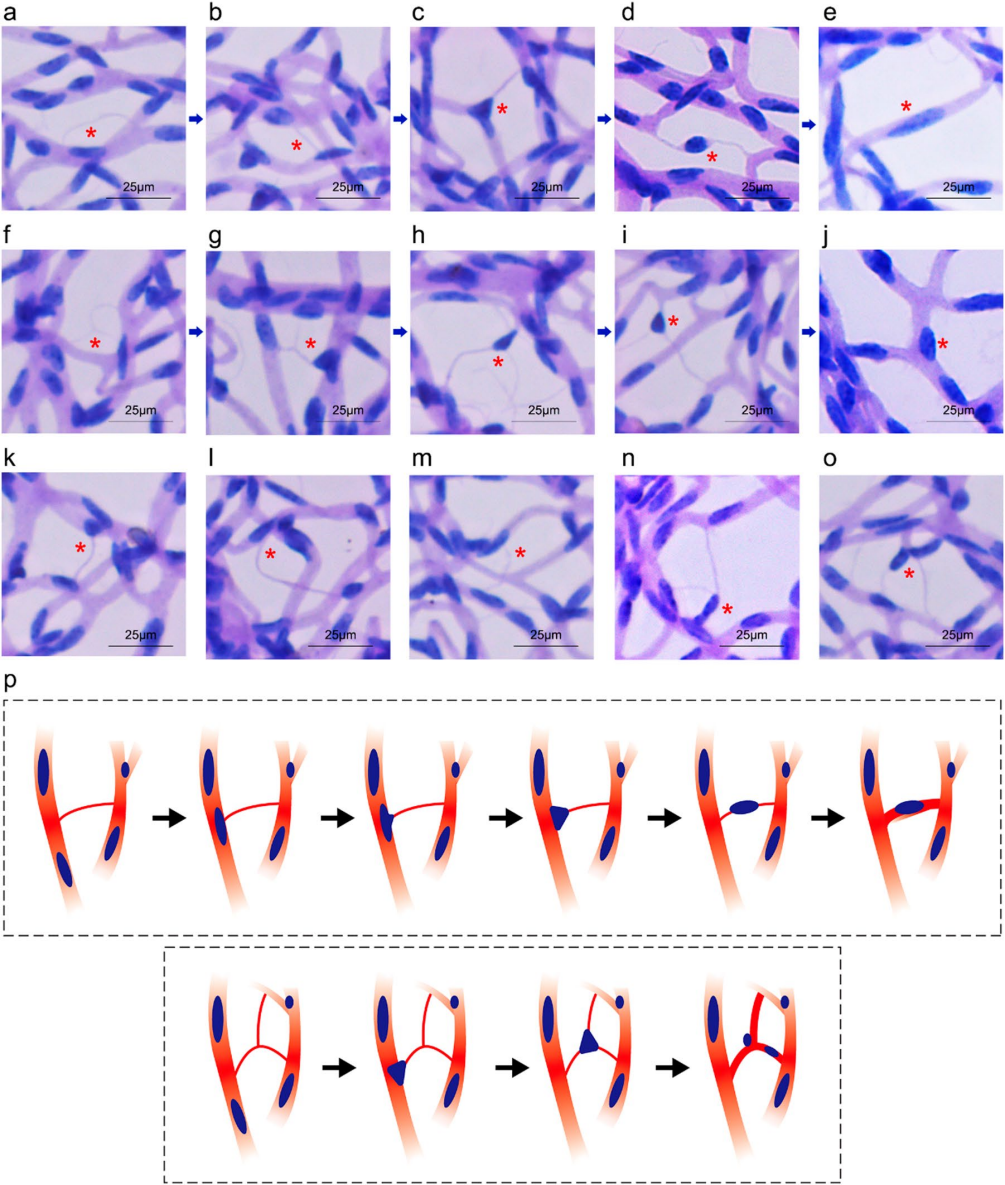
Representative morphologic appearances of string vessels/intercapillary bridge-like structures in P6d normal C57BL/6 mouse retina. **a**–**o** Representative high-magnification images from PAS-stained trypsin-digested retinal vasculature mounts of P6d normal C57BL/6 mice. Red stars indicate string vessels/intercapillary bridge-like structures. The panels show different static morphologic appearances, including string-like structures between two capillaries, string-like structures connecting vascular-associated nuclei, nuclear protrusion toward the string-like structure, and nuclei located near or within the string-like structure. **f**–**j** show representative three-way string-like connections among capillaries. **k**–**o** show additional morphologic patterns. These images were not obtained by time-lapse imaging or serial observation of the same vessel. Therefore, the linked panels should be interpreted as a putative morphologic sequence rather than direct evidence of temporal progression. **p** Schematic diagram showing a hypothetical model in which string vessels may participate in retinal capillary formation during vascular development. Further experimental validation is required to confirm this proposed process

Mounts of trypsin digestion of retinal vessels in mouse (S Fig. 4), rat (S Fig. 5), P17d mouse and OIR model mice (S Fig. 6) were displayed. The retinal vascular network was intact without damage, folding, or crimping (100×). The blood vessels at all levels were distributed, and there was no residual nerve fiber tissue between capillaries (200×). The distribution of capillaries and cell nuclear morphology was observed clearly (400×). It should be noted that, in OIR model mice, the central retina has an avascular area due to high oxygen, the inner limiting membrane closely adheres to the vascular network, and the visualization of blood vessels is weaker than that of normal mice.

### Intracapillary bridges and acellular capillaries identification

Features of acellular capillaries in a mouse model of diabetes: diameter greater than 20% of the diameter of adjacent capillaries; no nuclei anywhere along their length; fringes like pleated skirts; never seen in the normal retinal vascular network; they are residual vascular basement membrane residues after endothelial cells and pericyte death. Intercapillary bridges in normal mice: diameter less than 20% of the diameter of adjacent capillaries; usually connect nuclei, sometimes two or three capillaries; smooth edges; may be involved in angiogenesis during normal vascular development or under pathological conditions (Fig. 2).

### Intracapillary bridges may participate in angiogenesis during vasculature development and neovascularization in vascular retinopathy

The results of retinal fluorescence whole-mount stained with IB4 showed that the retinal vasculature development was a process that started from zero. There were no blood vessels in the retina at P0d. Then, blood vessels gradually grow from the retina’s center to the peripheral retina. The vascular network completely covered the entire retina at P7d (Fig. 3). P6d in the vascular development age group has the maximum quantity of string vessels. String vessel showed a decreasing trend at P6d, P2w, and P3w. String vessels in P3w, P6w, and P8w keep the same number and decrease in P24w. P17d OIR mouse model has more string vessel numbers than P2w and P3w (Fig. 4). P6d is in the developmental stage of retinal blood vessels. A large number of string vessels can be found in P6d mice, indicating that string vessels are likely to be involved in the development of retinal blood vessels. P7d OIR mouse model has more string vessel numbers than P2w and 3w, indicating that string vessels are likely to be involved in the angiogenesis in vascular retinopathy. Although string vessels are visible in both methods, the PAS-stained trypsin-digested retinal vasculature mount is more suitable for observing String vessels than the Retinal whole-mount + IB4 fluorescence staining. The shape and position of the nucleus can be observed in the PAS-stained trypsin-digested retinal vasculature mount (Fig. 5). Nuclear counterstaining was not performed in the IB4-stained retinal whole-mounts because these specimens retained the full retinal tissue; therefore, pan-nuclear staining would label nuclei from multiple retinal cell layers and obscure the specific assessment of vascular-associated nuclei. Nuclear morphology and nuclear position were therefore evaluated using PAS-stained trypsin-digested retinal vasculature mounts, in which nonvascular retinal tissue had been removed. Although some intercapillary bridge-like structures could be observed in IB4-stained retinal whole-mounts, IB4 labelling is not suitable for detecting all string-vessel-like structures, especially acellular capillaries lacking endothelial cells. Therefore, IB4 staining was used mainly to visualize the retinal vascular network, while PAS-stained trypsin-digested retinal vasculature mounts were used for evaluating acellular capillaries, intercapillary bridges, and nuclear morphology.

Figure 6 shows representative static morphologies of string vessels/intercapillary bridge-like structures observed in PAS-stained trypsin-digested retinal vasculature mounts from P6d normal C57BL/6 mice. P6d was selected because this stage corresponds to active postnatal retinal vascular development and showed the highest number of string vessels in our quantitative analysis. The observed morphologies included string-like structures between capillaries, string-like structures connecting vascular-associated nuclei, nuclei protruding toward the string-like structure, and nuclei located near or within the string-like structure. These images were not obtained by time-lapse imaging or serial observation of the same vessel. Therefore, they suggest a possible association between string vessels and capillary remodeling during retinal vascular development, but they do not directly demonstrate a sequential angiogenic process. Based on these static morphologic observations, we propose a hypothetical model in which string vessels may participate in retinal capillary formation.

## Discussion

In 1960, Kuwabara and Cogan [9] introduced trypsin-digested retinal vasculature mount preparation for the first time. After that, many researchers made improvements in the method [8, 22]; However, it was always difficult to maintain the integrity and repeatability of the retina. The retina of a mouse is smaller and more fragile than that of a rat. However, for the vascular retinopathy model, mice have the advantages of easy feeding and lower cost. Cho et al. [11] provided many valuable experimental skills and experience: ddH_2_O washing the retina after trypsin digestion is beneficial for retinal vascular network separation. All instruments separating the retinal vascular network should be pre-soaked with trypsin to prevent the vascular network from adhering to the device. Nevertheless, we ran into trouble separating the vascular network step. R. A. Cuthbertson and T. E. Mandel found that adjusting the enzyme concentration and digestion time could not entirely digest the neural and fibrous tissues attached to the retinal vascular network, and physical removal was required, which would lead to the tearing of the vascular network [23]. Meanwhile, glass pipette sucking and blowing cannot clear the residual nerve fiber tissue adhering to the vascular network. Suppose the retinal vascular network is directly clamped with non-toothed forceps out of the water. In that case, it is easy to form a tear in the vascular network at the clamping place, and the integrity of the vascular network cannot be maintained. The reason is that there is still much residual nerve fiber tissue attached to the vascular network, forming a specific gravity that can tear the vascular network. After many attempts, we found a solution: Using non-toothed micro forceps in the right hand, gently clamped the central part of the internal limiting membrane and vascular network to control the position of the retina, using a glass rod in the left hand under the retina to lift the retina out of the water surface, this procedure removes most of the nerve fiber tissue attached to the vascular network, preventing the tear of the vascular network caused by the overweight retina lifted out of the water surface and ensuring the integrity of the vascular network. If simply using a glass rod without non-toothed forceps, it can be challenging to control the position of the retina because the glass rod surface is too smooth. Since the small round glass rod does not adhere to the vascular network (its smooth glass surface), it is also an excellent instrument to help the vascular network unfold in water. In addition, some technologies are discussed: (1) Fixation: 4% paraformaldehyde-fixed time. Some literature has reported that fixed time does not affect retinal vascular mount digested by trypsin [9]. Some suggested fixing the eyeball for at least 24 h [24]. According to our experience, after removing the eyeball from the mouse, the retina was separated after fixation with 4% paraformaldehyde at room temperature for 48 h. Nevertheless, if the fixation time is too short, the retina may be too soft to separate. (2) Digestion time: We found that 3% trypsin solution was placed in a 37℃ water bath for 3–3.5 h for digestion. After digestion, the retina was rinsed in ddH_2_O for 5 min twice, 15 min once. This step facilitates the separation of the vascular network from the outer layer of the retina. (3) Mounting: a glass pipette coated with pancreatic enzyme, full of water, absorbs the retinal vascular network, quickly transferring the retinal vascular network onto the ddH_2_O droplet on the slide, and adjusting the vascular network with a glass rod. Through trial and technical improvement, we can successfully prepare a complete mouse retinal capillary mount repeatedly, laying a foundation for the experimental study of vascular retinopathy.

String Vessels of different weeks were observed and quantified. Kuwabara [9] describes the intercapillary bridge as a strand extending from one capillary’s nuclear region to another capillary’s nuclear region and having a nucleus adjacent to the capillary wall. We found that the number of string vessels in developing vasculature(P6d) was far higher than in mature vasculature(P6w). Researchers found numerous string vessels in newborn babies’ brains [19, 25]. Their observations in the brain are consistent with our observations in the retina. We found that retinal vasculature development in postnatal mice is a process from zero: The retina contains no blood vessels at P0. Then, blood vessels gradually grow from the retina’s center to the peripheral retina. The vascular network completely covered the entire retina at P7. This suggests that string vessels may be involved in developing blood vessels, especially capillaries. We also found more string vessels in the P17 OIR model mice than in normal mice. This suggests that string vessels may be involved in the formation of neovascularization under pathological conditions. Observing pericytes migrate along the string vessels made Pfister believe diabetic pericyte loss results from pericyte migration [26]. We observed pericytes migrate along the string vessels to the string vessel center, and endothelial cells migrate along the string vessels. Intercapillary bridges are fundamentally different from acellular capillaries: acellular capillaries in diabetic mice have the following characteristics: diameters more than 20% of the diameter of adjacent capillaries; Having no nucleus anywhere along its length. The edges of acellular capillaries are pleated; They are never present in the normal retinal vascular network. Formation may be associated with endothelial and pericyte death. Intercapillary bridges have the following characteristics: diameters less than 20% of the diameter of adjacent capillaries; Usually connect the nucleus, sometimes connect two or three capillaries; Smooth edge; May be involved in angiogenesis. Menes-Jorge’s study [20] showed that intercapillary bridge cells expressed NG2, PDGFR-β, CD34, and the tomato lectin, convincing him that intercapillary bridge cells were a specific pericyte subtype with the same characteristics as endothelial cells. Moreover, in the glaucoma model, intercapillary bridge cells and neovascularization increase in parallel [20].

The abundance of string vessels/intercapillary bridge-like structures in P6d normal mice suggests that these structures may be associated with active postnatal retinal vascular development. In addition, the increased number of string vessels in P17d OIR mice suggests that similar structures may also be associated with pathological vascular remodeling. However, the morphologic panels in Fig. 6 were obtained from fixed PAS-stained trypsin-digested retinal vasculature mounts of P6d normal mice and represent static observations rather than serial/time-course imaging of the same vessel. Therefore, the proposed role of string vessels in capillary formation should be interpreted as a hypothesis. Further studies using live imaging, lineage tracing, endothelial/pericyte markers, and basement-membrane markers such as Collagen IV or laminin are needed to determine whether these structures actively contribute to angiogenesis.

Overall, our observations suggest that string vessels/intercapillary bridge-like structures may be associated with retinal vascular development and pathological vascular remodeling. The representative morphologies shown in Fig. 6 were obtained from fixed PAS-stained trypsin-digested retinal vasculature mounts and therefore do not provide direct evidence of endothelial or pericyte migration, lumen formation, or a sequential angiogenic process. Based on these static morphologic findings, we propose that string vessels may participate in capillary remodeling or formation; however, this remains a hypothesis. Further studies using live imaging, lineage tracing, endothelial/pericyte-specific markers, and basement-membrane markers such as Collagen IV or laminin are needed to clarify the cellular composition and functional role of these structures.

A limitation of this study is that trypsin-digested retinal vasculature mounts, although useful for isolating the retinal microvasculature and observing vascular morphology, do not preserve the original three-dimensional retinal architecture or the spatial relationship between vessels, neurons, glial cells, and other retinal components. Therefore, the relationship between string vessels/intercapillary bridge-like structures and the surrounding retinal tissue could not be evaluated in this preparation. In addition, IB4-labelled retinal whole-mounts were not combined with basement-membrane or cell-specific markers. Because IB4 primarily labels endothelial-associated vascular structures, endothelium-free acellular capillaries or basement-membrane remnants may not be detected by IB4 staining alone. Challa et al. used anti-collagen antibody staining to study string vessels in three dimensions [19]. Therefore, future studies using retinal wholemount immunolabelling for basement-membrane components, such as Collagen IV or laminin, together with endothelial, pericyte, glial, and neuronal markers, would provide a more complete assessment of the cellular composition, three-dimensional structure, and neurovascular relationship of these string-vessel-like structures. In addition, this study did not include a formal quantitative comparison between the modified preparation method and conventional methods using predefined objective endpoints. Therefore, although the modified method appeared to improve retinal vascular network integrity and visualization of string-vessel-like structures, this methodological advantage should be further confirmed by standardized comparative studies with masked assessment. In addition, the distinction between acellular capillary like structures and intercapillary bridge-like structures was based mainly on qualitative morphology. Quantitative morphometric analysis, including diameter measurement, nuclear counts, and endothelial/pericyte-specific immunolabelling, is needed to validate these criteria.

## Supplementary Information

Below is the link to the electronic supplementary material.

Supplementary Material 1

## Data Availability

All data generated or analyzed during this study are included in this published article. Any additional data details can be requested from the corresponding author upon request.

## References

[1] Cheung N, Mitchell P, Wong TY. Diabetic retinopathy Lancet. 2010;376(9735):124–36. 20580421 10.1016/S0140-6736(09)62124-3

[2] Phelps DL. Vision loss due to retinopathy of prematurity. Lancet. 1981;1(8220 Pt 1):606. 6110833 10.1016/s0140-6736(81)92047-x

[3] Olivares AM, et al. Animal Models of Diabetic Retinopathy. Curr Diab Rep. 2017;17(10):93. 28836097 10.1007/s11892-017-0913-0 PMC5569142

[4] Connor KM, et al. Quantification of oxygen-induced retinopathy in the mouse: a model of vessel loss, vessel regrowth and pathological angiogenesis. Nat Protoc. 2009;4(11):1565–73. 19816419 10.1038/nprot.2009.187 PMC3731997

[5] Grossniklaus HE, Kang SJ, Berglin L. Animal models of choroidal and retinal neovascularization. Prog Retin Eye Res. 2010;29(6):500–19. 20488255 10.1016/j.preteyeres.2010.05.003 PMC2962694

[6] Kim SH, et al. Morphologic studies of the retina in a new diabetic model; SHR/N:Mcc-cp rat. Yonsei Med J. 1998;39(5):453–62. 9821795 10.3349/ymj.1998.39.5.453

[7] Ashton N. Studies of the retinal capillaries in relation to diabetic and other retinopathies. Br J Ophthalmol. 1963;47(9):521–38. 14189723 10.1136/bjo.47.9.521 PMC505844

[8] Laver NM, Robison WG Jr., Pfeffer BA. Novel procedures for isolating intact retinal vascular beds from diabetic humans and animal models. Invest Ophthalmol Vis Sci. 1993;34(6):2097–104. 8491560

[9] Kuwabara T, Cogan DG. Studies of retinal vascular patterns. I. Normal architecture. Arch Ophthalmol. 1960;64:904–11. 13755464 10.1001/archopht.1960.01840010906012

[10] Dietrich N, Hammes HP. Retinal digest preparation: a method to study diabetic retinopathy. Methods Mol Biol. 2012;933:291–302. 22893415 10.1007/978-1-62703-068-7_19

[11] Chou JC, Rollins SD, Fawzi AA. Trypsin digest protocol to analyze the retinal vasculature of a mouse model. J Vis Exp. 2013;(76):e50489. 10.3791/50489 PMC3913093 23793268

[12] Brown WR. A review of string vessels or collapsed, empty basement membrane tubes. J Alzheimers Dis. 2010;21(3):725–39. 20634580 10.3233/JAD-2010-100219 PMC3081641

[13] Wang H, et al. MicroRNA-93-5p participates in type 2 diabetic retinopathy through targeting Sirt1. Int Ophthalmol. 2021. 10.1007/s10792-021-01953-4 34313929

[14] Elshaer SL, et al. Modulation of p75(NTR) on mesenchymal stem cells increases their vascular protection in retinal ischemia-reperfusion mouse model. Int J Mol Sci. 2021;22(2). 10.3390/ijms22020829 PMC7830385 33467640

[15] Sorenson CM, et al. Thrombospondin-1 deficiency exacerbates the pathogenesis of diabetic retinopathy. J Diabetes Metab. 2013;Suppl 12. 10.4172/2155-6156.S12-005 PMC3818794 24224119

[16] Tien T, et al. Downregulation of Connexin 43 promotes vascular cell loss and excess permeability associated with the development of vascular lesions in the diabetic retina. Mol Vis. 2014;20:732–41. 24940027 PMC4043608

[17] Kim D, et al. Decreased lysyl oxidase level protects against development of retinal vascular lesions in diabetic retinopathy. Exp Eye Res. 2019;184:221–6. 31022398 10.1016/j.exer.2019.04.019 PMC6625661

[18] Cajal SR. Contribution a la connaissance de la nevroglia cerebrale et cerebeleuse dans la paralyse generale progressive.

[19] Challa VR, et al. A three-dimensional study of brain string vessels using celloidin sections stained with anti-collagen antibodies. J Neurol Sci. 2002;203–4:165–7. 10.1016/s0022-510x(02)00284-8 12417377

[20] Mendes-Jorge L, et al. Intercapillary bridging cells: immunocytochemical characteristics of cells that connect blood vessels in the retina. Exp Eye Res. 2012;98:79–87. 22484557 10.1016/j.exer.2012.03.010

[21] Tual-Chalot S, et al. Whole mount immunofluorescent staining of the neonatal mouse retina to investigate angiogenesis in vivo. J Vis Exp. 2013;(77):e50546. 10.3791/50546 PMC3732076 23892721

[22] Danis RP, Wallow IH. HRP/trypsin technique for studies of the retinal vasculature. Invest Ophthalmol Vis Sci. 1986;27(3):434–7. 3949471

[23] Cuthbertson RA, Mandel TE. Anatomy of the mouse retina. Endothelial cell-pericyte ratio and capillary distribution. Invest Ophthalmol Vis Sci. 1986;27(11):1659–64. 3771146

[24] Huang Q, et al. PEDF-deficient mice exhibit an enhanced rate of retinal vascular expansion and are more sensitive to hyperoxia-mediated vessel obliteration. Exp Eye Res. 2008;87(3):226–41. 18602915 10.1016/j.exer.2008.06.003 PMC2562453

[25] Binswanger O, Schaxel J.J.A.F.P.U N. Beiträge zur normalen und pathologischen Anatomie der Arterien des Gehirns. 1917;58(1):141–87.

[26] Pfister F, et al. Pericyte migration: a novel mechanism of pericyte loss in experimental diabetic retinopathy. Diabetes. 2008;57(9):2495–502. 18559662 10.2337/db08-0325 PMC2518502

