# Supplementary material for: Modified skills of trypsin-digested retinal vasculature mount preparation and string vessels observation
Source: BMC Ophthalmol. 2026 May 25;26:438. doi: 10.1186/s12886-026-04951-1 (PMC13404314; doi:10.1186/s12886-026-04951-1)
Supplement: Supplementary file 1 — Supplementary Material 1 [file 12886_2026_4951_MOESM1_ESM.docx]

**S Fig.1** **Critical apparatus and instruments for the experiment a** C57/BL6 mouse. **b.** Shaker and 24-well plate. **c** Constant temperature water bath. **d** Anatomical microscope. **e** From left to right: curved micro scissors, non-toothed micro forceps *2, plastic Pasteur pipette which pipe orifice has been enlarged (used for transferring retina), glass pipette, glass rod, toothed micro forceps, sharp tip surgical blade, tissue scissors, and non-toothed curved forceps.

**S Fig.2 Typical retinal images after digestion, ddH_2_O washing, or residuals thoroughly cleared** **a** Image after trypsin digestion: the outer layer of the retina became flocculent. **b** Image after the first ddH_2_O washing: a small part of the outer layer was moved from the retina. **c** Image after another two ddH_2_O washing: the retina became transparent, but many fibral neural tissues were still left between the retinal vasculature networks. **d** Image of the clean retinal vascular network before mounting.

**S Fig.3 Schematic diagram of critical operation skills** Blue arrow: glass rod; Black arrow: non-toothed micro forceps; Yellow arrow: internal limiting membrane; Green arrow: retinal vascular network; Orange arrow: curved micro scissors. **a-c** Non-toothed micro forceps were used to gently clamp the central part of the internal limiting membrane to control the position of the retina, and a glass rod under the retina was used to raise the vascular network higher than the water surface and quickly return into the water. **d-e** Repeatedly move the retinal vascular network up and down the water's surface with non-toothed micro forceps alone. **f** Cut the internal limiting membrane using the tip of the curved micro scissor when the vascular network becomes clean.

**S Fig.4 Retinal vasculature mount of the mouse** **a** The retinal vascular network was intact without damage, folding, or crimping (100×). **b** The blood vessels at all levels were distributed, and there was no residual nerve fiber tissue between capillaries (200×). **c** The distribution of capillaries and cell nuclear morphology.

**S Fig.5 Retinal vasculature mount of the rat a** The retinal vascular network was intact without damage, folding, or crimping (100×). **b** The blood vessels at all levels were distributed, and there was no residual nerve fiber tissue between capillaries (200×). **c** The distribution of capillaries and cell nuclear morphology.

**S Fig.6 Retinal vasculature mount of p17 OIR C57/BL6 mouse a-c** Normal model. Although P17 mouse' retina was tough to handle, a clean and complete retinal vasculature mount was still aquireble using the modified method. **d-f** Oxygen induced retinopathy model. The internal limiting membrane of OIR model mice was thin, brittle and adhered to the retinal vascular network, so it was difficult to separate, and the central retina was vascularless. Despite careful handling, there are remnants of the inner boundary membrane and nerve fiber tissue.

**S Fig. 7. Representative technical problems encountered during unmodified or suboptimal trypsin-digested retinal vasculature mount preparation.**
a Residual neural fibrous tissue or internal limiting membrane remnants remaining between the retinal vascular networks. b Poor visualization of endothelial-cell nuclei after hematoxylin staining. c Incomplete unfolding of the retinal vascular network. These images are shown as representative examples of possible technical problems and do not indicate the frequency, severity, or inevitability of failure during retinal vasculature mount preparation.

**
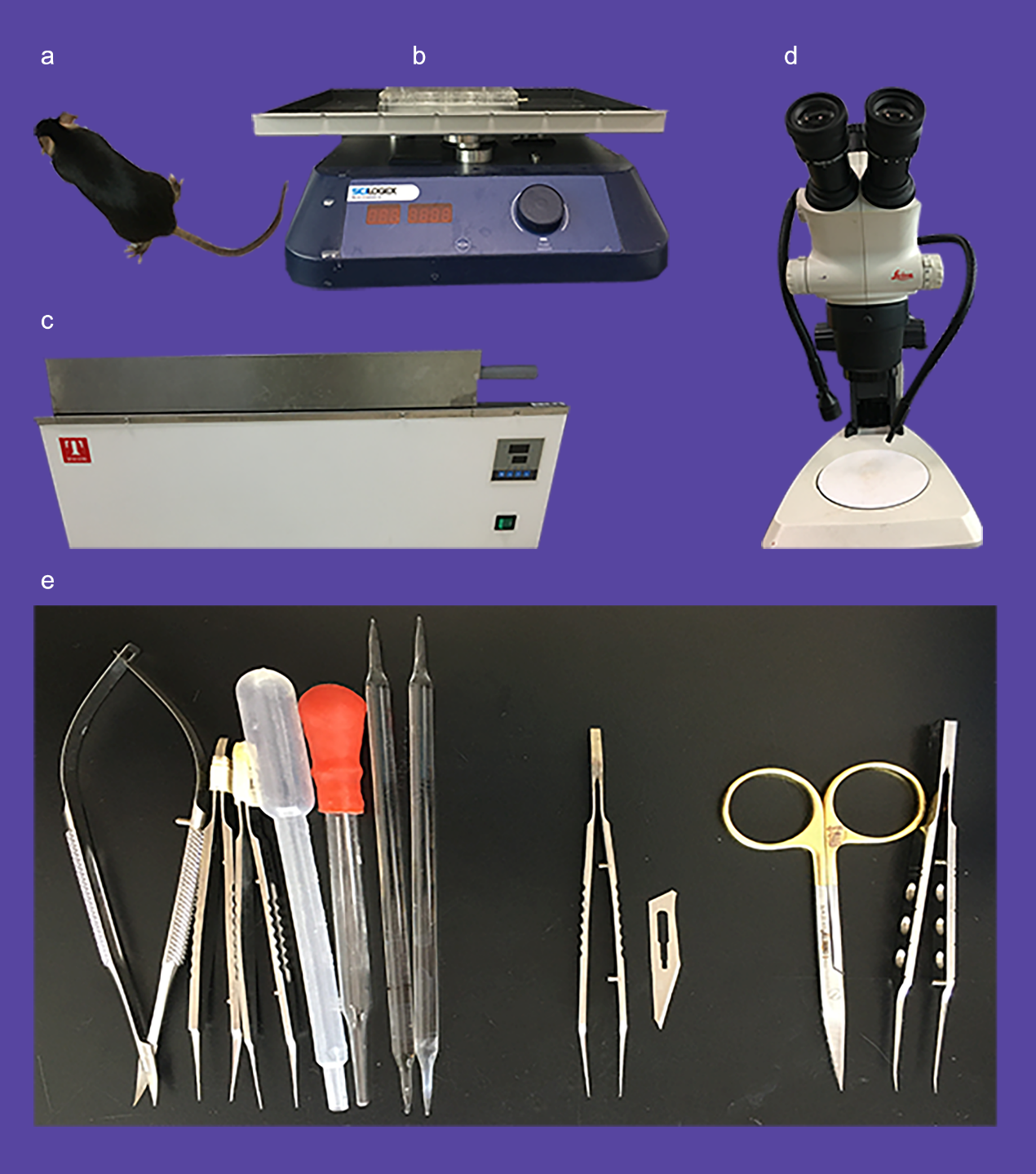
Fig.1**

**
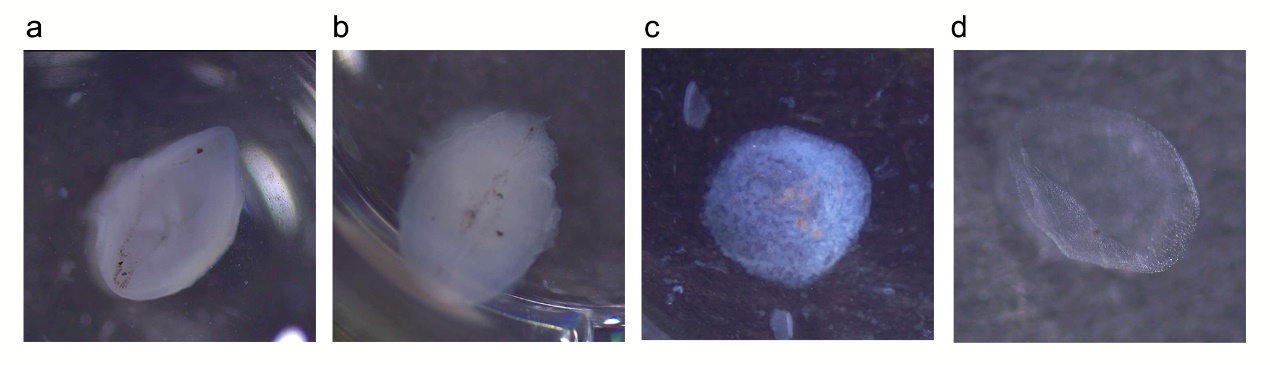
Fig.2**

**Fig.3**


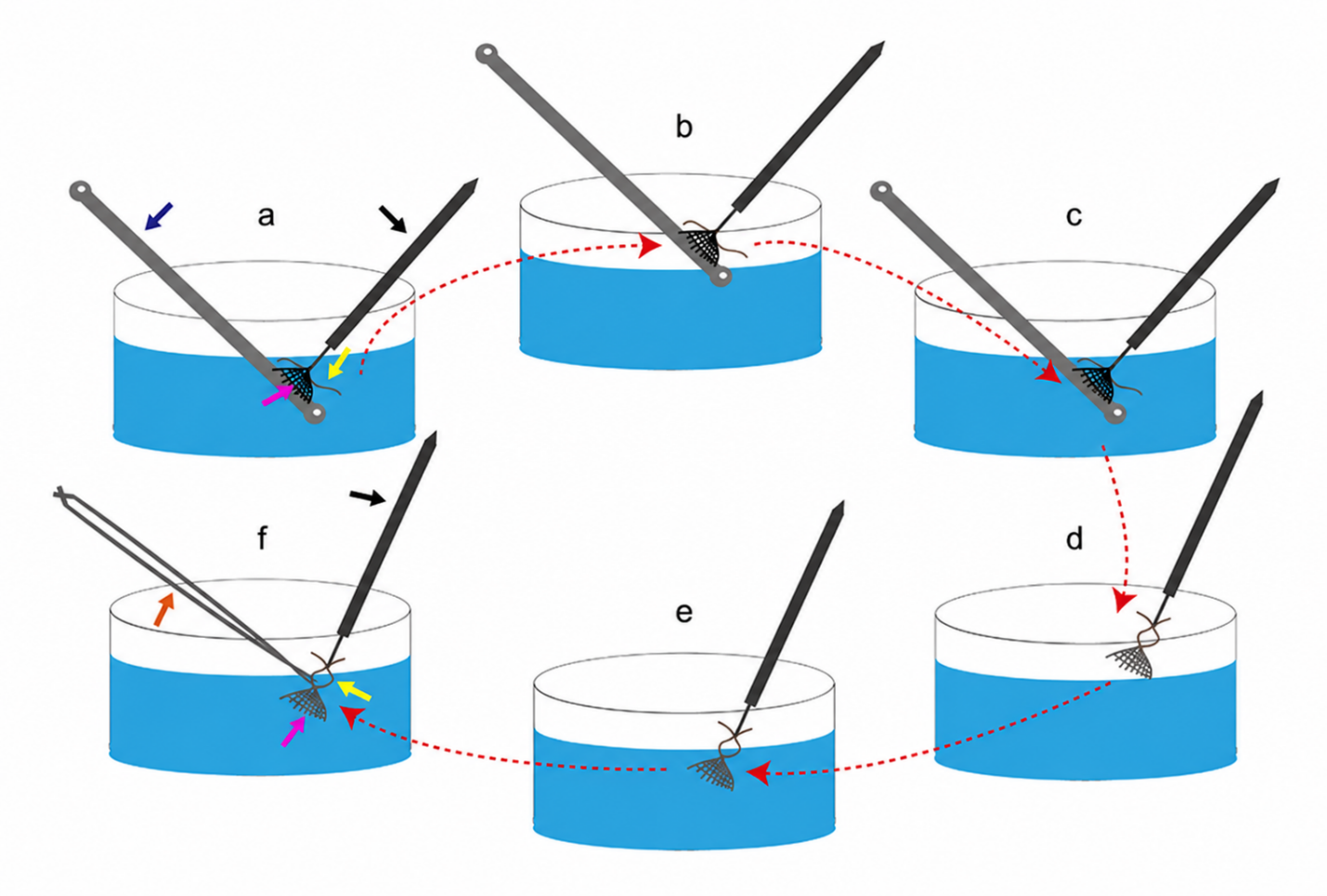


**
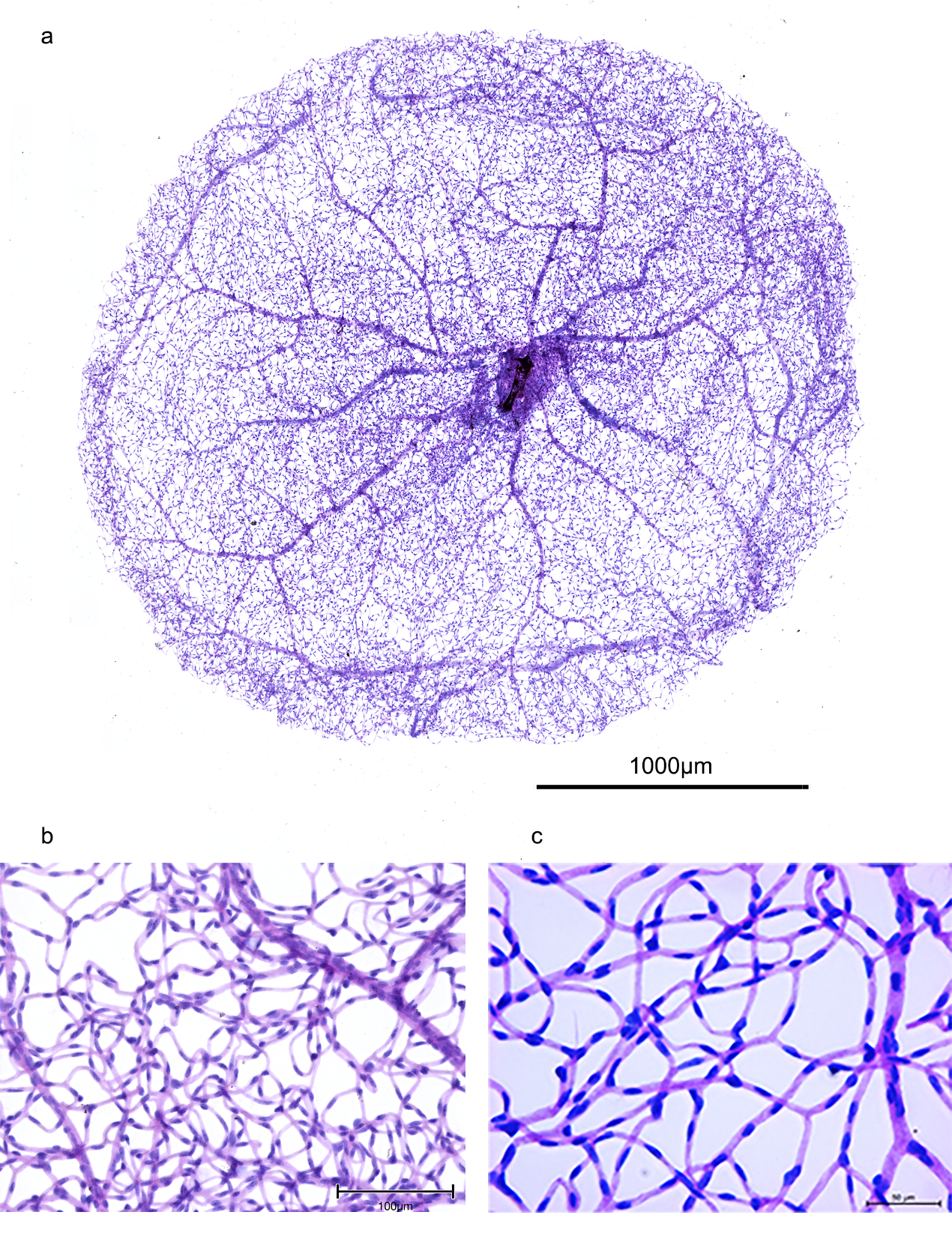
Fig.4**

**
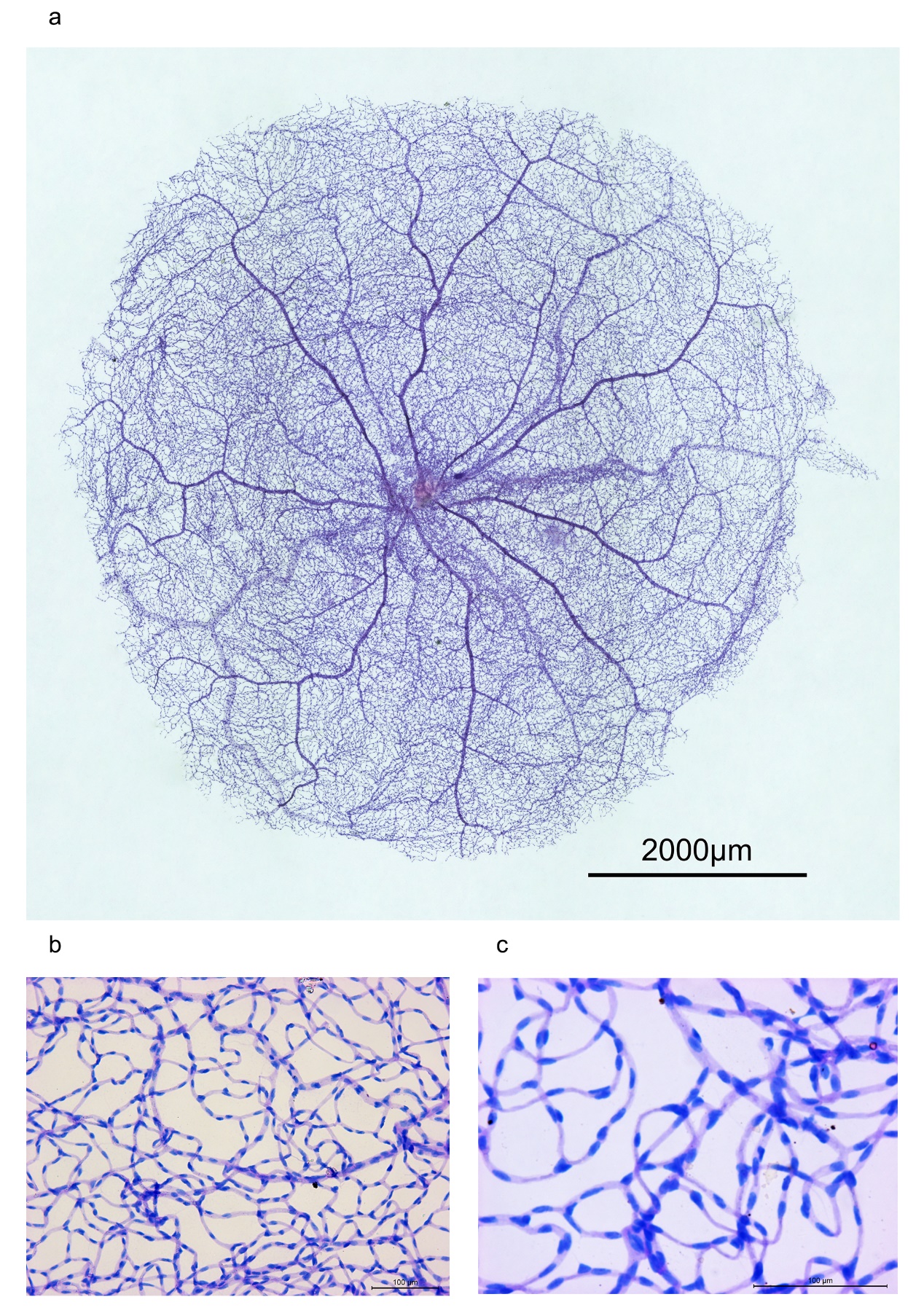
Fig.5**

**
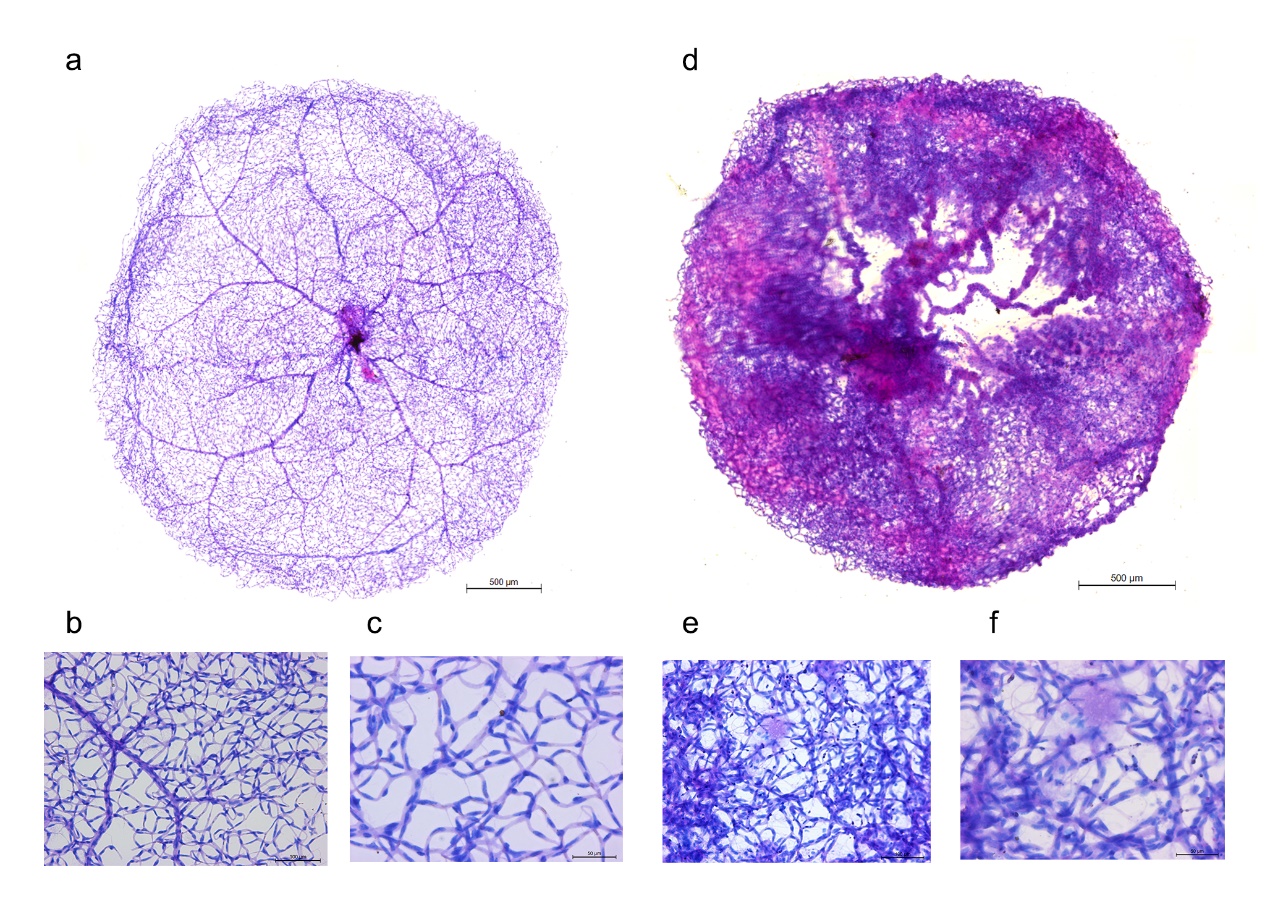
Fig.6**

**
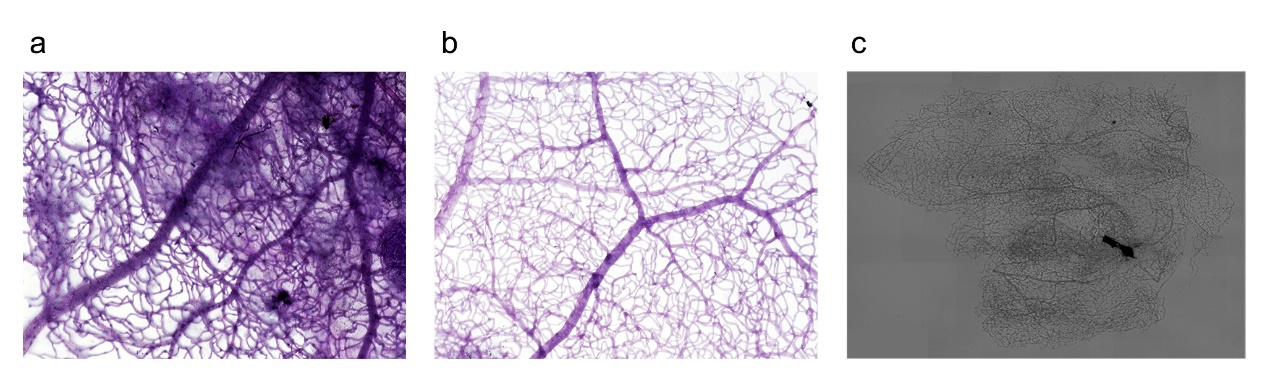
Fig.7**
